# Septicemia and the Curette

**Published:** 1903-02

**Authors:** H. Plympton

**Affiliations:** Brooklyn, N. Y.


For Texas Medical Journal.

### Septicemia and the Curette.

BY H. PLYMPTON, M. D., BROOKLYN, N. Y.

To attempt to break up an old established custom in any line of life is at best, a thankless job, and one likely to call down harsh criticism upon the head of the daring iconoclast.

To attempt to uproot old prejudices existing in favor of a certain line of practice in surgery, and diametrically oppose such practice, is to invite from some, adverse criticism of the harshest kind. The only recompense for this is a logical refutation of, or concurrence in the argument advanced, on the part of other members of the profession.

This latter is what I hope for, and if I provoke a discussion, or start a line of thought in the minds of half of the readers of this article, I shall have achieved all I started out to do.

Curetting the uterus to remove fragments of after-birth or other debris has been taught in the medical schools from time immemorial, and it is firmly fixed in the receptive and retentive mind of every medical student that the first move following and such abnormal uterine condition, is to cleanse the uterus by means of the curette.

That the organ should be thoroughly and aseptically cleansed admits of no argument, but that the work should be done with the curette, I deny most emphatically.

The majority of cases of death following the decomposition of fœtus or placenta in utero, are caused by the use of the curette, and I hold that septicemia may be avoided if a more rational procedure be resorted to.

The condition of the uterus containing septic matter is one of great congestion; the thickened walls being coated internally and over the os with a thick, brown, tenacious mucus.

The congestion is active, and therefore the more dangerous in the event of the admission of septic matter into the circulation.

If the curette is used, denuding the walls of their protective covering, an immediate vaccination takes place with a septic virus, septicemia following in an incredibly short space of time (chemical metamorphosis is marvelously rapid in the circulatory system) and death quickly ensues.

If without using the curette, we can remove the septic matter from the uterus without disturbing the mucus covering, and enable the uterus of itself to exepl the coating, we shall have taken a long step forward in the treatment of this class of uterine cases.

The uterus, by reason of its congestion may be made to perform a self-cleansing act by exciting the exudation of the serum of the blood into its cavity, thereby washing itself out, and expelling all septic matter instead of absorbing it.

This process of exosmosis is induced by a properly combined alkaline solution at a temperature of 100° and a strict avoidance of bichloride, carbolic acid, formaldehyde, or any antiseptic of an acid reaction or astringent nature, which would coagulate the fibrine and albumen of the blood.

The method of procedure is as follows:

First. The gentle removal of whatever fragments are lying in the uterine cavity, by means of forceps, care being taken not to tear from the walls the adherent piece.

Second. The gentle flushing of the uterine cavity with the alkaline solution (100°), the reservior containing the fluid being not more than two feet above the level of the hips.

If the flushing could be continuously administered for a few hours (say two or three), the conditions would be more speedily reduced to normal, but the discomfort of the position of the patient (on a douche pan) prevents this, and a flushing once every two hours with one quart of solution is about the limit of the treatment.

For flushing the uterus, I use a small dilating uterine douche, and as there is plenty of room for the escape of fluid and fragments, there is no danger of fallopian colic or salpingitis.

The first flushing is frequently followed by contractile pains and expulsion of any previously adherent pieces, together with much of the mucus.

A tablet of extract cannabis indica, gr. ¼; extract ergotin, gr. ½, every hour till desired effect is produced will contract uterus and alleviate pain.

The bowels should be moved freely, both by enema and cathartics.

During the interval between douches, the patient should be kept on her back with the hips sufficiently raised to permit the reaction in the vagina of as much of the alkaline solution as it will hold.

The rapidity with which this treatment will reduce temperature, relieve pain, stop vomiting and remove offensive odor is marvelous to one who has not tried it. Sometimes two flushings are sufficient to cleanse the uterus thoroughly; vaginal douches being all that are needed subsequently to complete the work.

Uterine congestion is speedily relieved, and the uterine discharge changes from brown, thick bad smelling mucus, to a thin transparent one, accompanied or followed by more or less of a flow of blood.

A reduction in the frequency of the flushings is desirable as soon as a tendency to return to normal conditions begins to be observed, as it frequently will return within twenty-four hours. Then simple vaginal douches every three hours with an occasional uterine flushing if symptoms indicate it.

The action of exosmosis (and endosmosis, for there is every reason to believe in the absorption of some of the fluid) is what is desired to relieve the existing congestion, as in a bronchitis, pneumonia, congestion of kidney, congestion of any mucous membrane, etc., and is the most rational means of restoring to normal condition.

I do not wish to be understood as decrying the use of that most valuable instrument, the curette, but only the abuse of it, towit: its employment under such conditions as make it practically a sharp weapon loaded with septic matter, dangerous beyond the poisoned arrow of the Malay, or the fang of the cobra, and utterly opposed to our modern ideas of antisepsis.

